# Fiscal and monetary policies: the cutting edge of advocacy and research on population health and climate change

**DOI:** 10.1177/17579139211059983

**Published:** 2021-11-24

**Authors:** Y Naik, A Brook, J Perraton, P Meier

**Affiliations:** The University of Sheffield and Leeds Teaching Hospitals NHS Trust, Sheffield, UK; The University of Sheffield and Leeds Teaching Hospitals NHS Trust, Sheffield, UK; The University of Sheffield, Sheffield, UK; University of Glasgow, Glasgow, UK

*This article outlines the likely mechanisms through which fiscal and monetary policies affect health and the environment, summarising innovative policies that may hold promise for planetary and population health.*

## Introduction

The recent pandemic, along with the pressing challenges of climate change and biodiversity loss, has led to increased recognition of the need for new forms of economic policy that prioritise people’s health and the environment.^1^ Macroeconomic policy includes fiscal and monetary policy.^2^ Fiscal policy involves choices around government revenue and spending and the balance between the two. Monetary policy includes setting interest rates and purchasing government securities or other assets. A wide range of such policies have been deployed following COVID-19.^3^

This article advocates complexity modelling as an innovative approach to study these policies given the multiple relevant mechanisms of effect. It then draws conclusions for future research priorities and public health action.

## How Fiscal and Monetary Policy Affect Population Health and Climate Change

Fiscal and monetary policy can significantly affect population health and environmental outcomes, for example through their influence on economic growth, which is often associated with improvements in population health.^4,5^ Beneficial effects of economic growth are thought to be due to increased government investment in services and infrastructure that promote good health, as well as increases in employment opportunities and household income. However, economic growth is currently also a driver of climate change and biodiversity loss, both of which have negative implications for population health and for economic growth itself.^1,6^ There is a substantial debate about the ongoing focus on economic growth, including whether there may be limits to this growth,^7^ or whether it is possible to ‘decouple’ it from resource use and carbon emissions.^8^

Fiscal and monetary policies may also affect health and health inequalities through their impacts on other macroeconomic factors such as inequality and poverty.^9^ There are complex relationships between these various macroeconomic factors, and their collective influence on health outcomes has not been robustly conceptualised or extensively studied. More direct mechanisms include changes in consumption such as reduced fossil fuel use and concomitant air pollution due to carbon taxes.^10^

## Innovative Fiscal and Monetray Policies

Many innovative fiscal and monetary policies have been proposed to address climate change.^11^ These include reducing subsidies to fossil fuel companies, or central banks reallocating resources to sustainable economic sectors. The Green New Deal is a combination policy designed to address climate change and social inequality through government investment in a greener and more equal society with a focus on good jobs.^12^ The Green New Deal is one example of such innovative policies that has been gathering support from health advocates.^13^

There is no integrated view of how these different fiscal and monetary policies influence health and environmental outcomes that takes into account the distinct and overlapping mechanisms of effect. It is therefore not currently possible to develop a robust appraisal of the likely health impacts of these innovative policies, or to assess how individual policies or combinations of them might result in synergies or trade-offs across health and environmental outcomes.^14^

**Figure 1:**
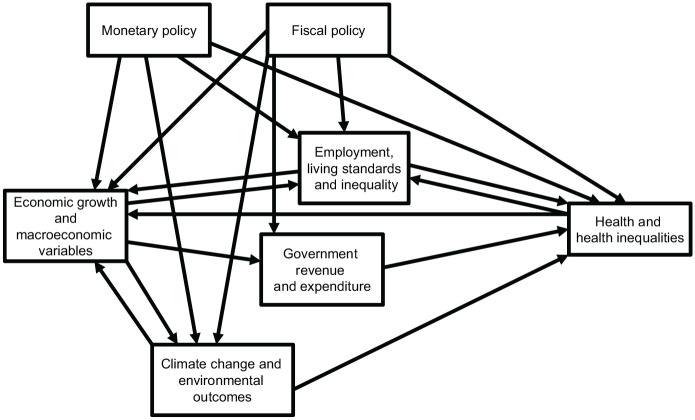
Illustrative conceptual model

An illustrative conceptual model summarising some of the mechanisms and policies described above is provided in Figure 1.

## Complex Systems Modelling?

The large number of dynamic relationships and feedback loops linking the economy, population health and environmental outcomes points to these forming part of a complex system.^15^ Complex systems require specific research methods, as they are characterised by nonlinear behavioural dynamics – for example stable states and tipping points (where the system undergoes a sudden shift) or emergence (where the behaviour of the whole system is qualitatively different from the behaviour of its individual components, and therefore whole system behaviour cannot be predicted from studying only the individual parts).

Given the wide range of relevant variables, limited uptake of key policy proposals and the urgent nature of environmental issues, modelling is an ideal strategy to assess the likely impacts of innovative fiscal and monetary policy to aid the further development of policy priorities and proposals. Modelling is particularly able to test a wide range of assumptions when there is uncertainty – as there is in this case.^14^

Past models have incorporated the relationships between the economy, the environment and determinants of health such as employment or inequality but to date these models have not considered health outcomes or health inequalities.^16^

## Implications

Achieving greater clarity on the likely health impacts will require collaboration across disciplines. While the urgency of climate change means we cannot wait for perfect evidence, we argue that increased understanding about the potential health impacts of monetary and fiscal policies is necessary to help steer policy as it develops. This will only be achieved if research funders prioritise this topic. It will also require interdisciplinary collaborations between public health, economics and environmental scientists. This article has made a case for complex systems modelling as a viable methodological approach for addressing these questions, though it is clear that there is also a need for more social epidemiology that can illuminate the relationships between the diverse variables in question and be used to populate such complex models. Such models can and should also be used to connect with public conversations about shared values that will shape trade-offs and decisions as we build a fairer, greener society and economy.

As health advocates, we should be clear about the evidence base for our policy demands. We should also be transparent about ethical trade-offs between the quality of evidence, levels of uncertainty and the urgent need for action. It seems clear that no single policy can solve climate change and health inequalities, requiring the adoption of a broad portfolio of well-aligned fiscal and monetary policies.

Public health agencies will also have a key role to play by working with key government departments such as finance ministries and central banks to embed health and wellbeing at the heart of fiscal and monetary policy.
